# Incidence of Atrial Fibrillation in Postmenopausal Women with Endometrial Cancer

**DOI:** 10.3390/jcm10020266

**Published:** 2021-01-13

**Authors:** Mariana S. Parahuleva, Julian Kreutz, Gerhild Euler, Dora Terzieva, Amar Mardini, Ekaterina Uchikova, Nikoleta Parahuleva

**Affiliations:** 1 Internal Medicine/Cardiology and Angiology, University Hospital of Giessen and Marburg, 35043 Marburg, Germany; Amar.Mardini@uk-gm.de; 2 Institute of Physiology, Justus Liebig University Giessen, 35390 Giessen, Germany; gerhild.euler@physiologie.med.uni-giessen.de; 3 Department of Clinical Laboratory, Medical University of Plovdiv, 4002 Plovdiv, Bulgaria; terzieva2006@yahoo.com; 4 Department of Obstetrics and Gynecology, Medical University of Plovdiv, 4002 Plovdiv, Bulgaria; euchikova@yahoo.com (E.U.); n_nikoleta1986@abv.bg (N.P.)

**Keywords:** endometrial cancer, atrial fibrillation, adiponectin, obesity, menopause

## Abstract

Endometrial cancer (EC) has been associated with an increased risk of cardiovascular disease, including atrial fibrillation (AF). We performed a prospective, case-controlled analysis among 310 Bulgarian women with new-onset, histologically confirmed EC, free of AF at the baseline survey, and women with normal (senile) endometrium/endometrial hyperplasia as a control group (*n* = 205). The risk of AF as well as relationship of adiponectin (APN) and high sensitivity C-reactive protein (hs-CRP) levels with AF in women with EC were calculated by Cox proportional hazards models. During the mean follow-up of 2.5 ± 0.5 years, new-onset AF had occurred in 11.7% of women with EC vs. 5.8% in the control group (*p* < 0.01). The risk of AF was highest in the first 6 months after new-onset EC, with an incidence rate ratio (IRR) of 1.19 (95% CI 1.10–1.29; *p* = 0.01). Women with EC, who were obese (body mass index (BMI) > 30 kg/m^2^) and younger (age < 60) were found to be more likely to develop AF (HR 1.95; 95% CI 1.18–3.32; *p* = 0.05). APN levels were not significantly associated with new-onset AF (95% CI 0.87–1.21; *p* = 0.063). However, the secondary analysis showed evidence of APN–AF association when adjusted for BMI (2.05; 95% CI 1.04–4.04; *p* = 0.037). We conclude that EC was significantly associated with the incidence of AF.

## 1. Introduction

Atrial fibrillation (AF) is the most common cardiac arrhythmia in clinical practice [1,2]. The occurrence and development of AF are associated with changes in the atria’s electrical properties and structure, known as electrical and structural remodeling [1,2]. Population-based studies have described risk factors for AF, including age, diabetes, and hypertension [2,3]. More recently, body mass index (BMI), natriuretic peptides, markers of inflammation, and coagulation markers have emerged as independent determinants, which may affect AF-related thromboembolic events [4,5,6]. Although numerous risk factors contribute to the development of AF, obesity—which leads to left atrial (LA) remodeling by various mechanisms—and metabolic risk factors loom as increasingly prominent contributors to cardiac dysrhythmia and are associated with a marked increase in the risk of developing AF [7,8].

Recent studies suggest that patients with non-cardiovascular diseases such as cancer also face a substantial risk of AF [9,10,11,12]. Interestingly, women with AF are at a higher risk of stroke and death than men and a higher risk of malignant cancer beyond one year of AF diagnosis [8,9,13]. Identification of novel factors influencing the development of AF is critical to the understanding and future prevention of the disease. In particular, a new study has provided yet more evidence that survivors of endometrial cancer (EC) should be closely monitored for cardiovascular disease [14]. These women are at a higher risk for various long-term cardiovascular problems than their cancer-free counterparts, especially phlebitis and thrombophlebitis, pulmonary heart disease, hypotension, and AF [14]. Furthermore, EC is the most common gynecological malignancy of the female reproductive tract and obesity is a major risk factor for EC [15,16,17,18]. Adipose tissue is an active endocrine organ that releases a number of cytokines and hormones, collectively termed adipocytokines, including adiponectin (APN), leptin, resistin, and tumor necrosis factor-alpha [15,16,17,18,19]. The adipocyte-derived hormone APN plays an important role in the development of obesity-related EC, as its levels decrease with higher adiposity and adipose-tissue inflammation [15]. This raises the possibility that decreased circulating levels of APN could help precipitate changes to the myocardial substrate necessary for the development of AF [19].

Both the incidence and prevalence of AF in Bulgaria are substantial and are likely to increase in the future [20]. Despite efforts to prevent AF-related morbidity and mortality, AF remains associated with an increased risk of heart failure, stroke, and death [21]. However, a better understanding of the mechanisms by which these risk factors, cardiovascular and non-cardiovascular, are associated with AF is necessary to inform novel preventative and therapeutic interventions. To our knowledge, EC has not been studied as a potential predictor of the incidence of AF in postmenopausal women. Therefore, the current study was designed to explore the association between EC and new-onset AF in a prospective cohort of 310 postmenopausal Bulgarian women who were followed up for 2.5 ± 0.5 years. The study also analyzed the effects of circulating APN and BMI on the independent association, which may constitute potential mediating mechanisms.

## 2. Materials and Methods

### 2.1. Study Design, Case Definition, and Control Selection

**Figure 1:**
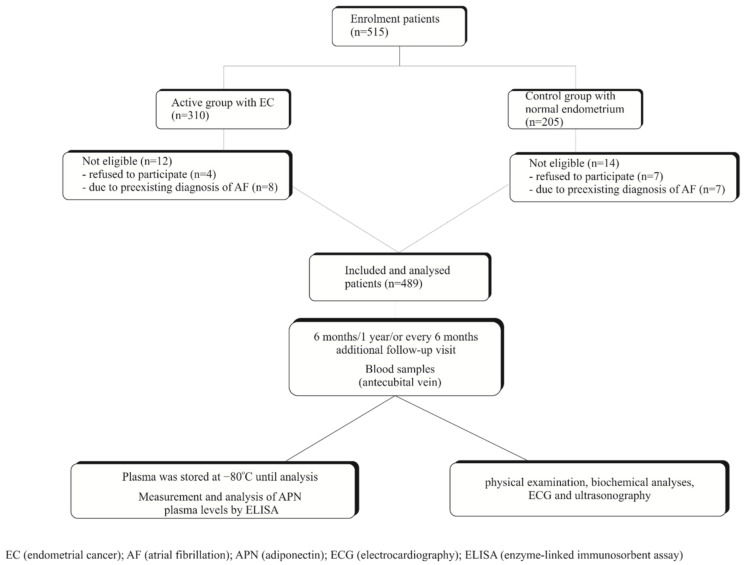
Flow chart of the study population.

This study is a monocentric, prospective case-control trial and included 515 postmenopausal Bulgarian women with vaginal bleeding (Figure 1) admitted to the Department of Obstetrics and Gynecology, University Hospital, Plovdiv, Bulgaria, between August 2014 and September 2017. Depending on the result of histology after curettage or hysteroscopy, 285 (92%) were identified as type I EC (all endometrioid adenocarcinomas) and 25 (8%) were identified as type II EC (papillary adenocarcinoma (*n =* 15) and serous adenocarcinoma (*n =* 10)). Thus, 310 individuals with histologically confirmed EC who were willing to participate in the study were selected as case group members. The controls were selected through frequency-matching from among individuals who tested for non-malignant endometrial tissues, which were also evaluated histologically by a pathologist and consist of normal (senile) endometrium (*n =* 161) and endometrial hyperplasia (*n =* 44). Specifically, candidate controls were stratified in accordance with age and other covariates of the case group and selected through convenience sampling. Additionally, a stratification analysis of the participants was carried out in two groups (51–65 years and >66 years). Finally, 205 controls were selected.

Cases and controls were excluded in cases of (i) previous history of cancer, (ii) polycystic ovary syndrome, (iii) deep vein thrombosis, (iv) pulmonary embolism, (v) acute myocardial infarction, (vi) recent percutaneous coronary intervention, (vii) AF, (viii) mitral regurgitation, (ix) heart failure (HF), (x) peripheral vascular disease, (xi) hematological disease, (xii) autoimmune disorders, and (xiii) unwillingness to provide written informed consent to participate. All women were free of infection at the time of blood collection. Blood pressure and the other traditional cardiovascular risk factors, including a lipid profile, were assessed, and this information is recorded in Table 1.

### 2.2. Ethical Statement

The study protocol and all experimental protocols were approved by the Ethics Committee of the Medical University of Plovdiv, Bulgaria, and informed written consent was obtained from all subjects after being informed of the research subject matter. All participants were assured that their personal information would be kept private. Each participant had the right to withdraw from the study at any time. Furthermore, all methods were carried out in accordance with the latest version of the Declaration of Helsinki and related notifications for the protection of human subjects.

### 2.3. Laboratory Analyses

All participants underwent a venipuncture at the morning before surgery after fasting overnight and without taking any hormonal therapy. Blood samples (35 mL) were placed in heparin-containing tubes. The plasma was separated from the blood cells using centrifugation at 800× *g* Relative Centrifugal Force (RCF) for 30 min at 4 °C, and the separated plasma was kept at −80 °C until use. The total time between the blood draw and freezing the plasma was no more than 4 h at all recruitment sites. Total circulating APN concentration was measured with a commercial immunoassay (Biovendor, Czech Republic) and expressed as ng/mL. Concentrations were calculated according to the standard concentrations and the corresponding optical density value at 450 nm. Intraassay and interassay coefficients of variation were 4% for APN.

Routine blood chemistry including high-sensitivity C-reactive protein (hs-CRP) levels and lipids (total cholesterol, low-density lipoprotein (LDL), high-density lipoprotein (HDL) cholesterol, and triglycerides) were analyzed using fresh blood samples according to established enzymatic methods at the local laboratories.

### 2.4. Follow-Up and AF Assessment

Patients were followed up at every 6 or 12 months routine medical examination until 30 September 2017, or until new-onset AF or death. Diagnosis of AF was made using 3 different approaches: a standard 12-lead electrocardiogram (ECG) as performed during the study exams, hospital discharge diagnoses, and death certificates. It was classed as new-onset if there was no prior diagnosis of AF. Within 2 years of AF onset, the most severe AF pattern was used to classify women as having paroxysmal or non-paroxysmal AF [21]. Paroxysmal AF was defined as self-terminating AF, lasting <7 days that did not require cardioversion [21].

### 2.5. Echocardiography

Two-dimensional and Doppler echocardiography was performed by an experienced sonographer. Left ventricular end-diastolic diameter (LVEDD) and left atrium (LA) dimensions were standard M-mode measurements, as recommended by the European Association of Echocardiography [22]. Left ventricular ejection fraction (LVEF) was calculated using the modified Simpson’s rule [22].

### 2.6. Assessment of Potential Covariates

Patients’ demographic information and clinical characteristics of patients, including age, body weight, alcohol use, smoking, prevalent health conditions, and medication usage, were recorded at baseline, using patient self-report and an interview-administered standardized questionnaire (Table 1). Smokers were defined as women who smoked 10 or more cigarettes per day. BMI was calculated by dividing body weight in kilograms (kg) by the square of a patient’s height in meters (m^2^). The patients’ BMI was categorized as normal (18.5 to 24.9 kg/m^2^), overweight (25 to 29.9 kg/m^2^), and obese (>30 kg/m^2^). Hypertension was defined by blood pressure ≥140 mm Hg systolic or ≥90 diastolic or self-reported and anti-hypertensive treatment. Diabetes was defined by fasting glucose ≥126 mg/dL or hypoglycemic therapy and hyperlipidemia (total cholesterol concentration ≥240 mg/dL, fasting triglyceride concentration ≥150 mg/dL) or receiving hyperlipidemia medication. Prevalent health conditions were assessed and recorded (Table 1).

### 2.7. Endpoints and Collection of Data

The primary study endpoint was a diagnosis of paroxysmal and non-paroxysmal AF. The cohort and the background population were followed for the endpoint until 30 September 2017.

### 2.8. Statistical Analysis

Data were found not to be normally distributed according to the D’Agostino and Pearson omnibus normality test. Continuous variables were described as mean ± standard deviation or median and compared using the Kruskal–Wallis test followed by Dunn’s corrections for multiple comparisons. Categorical variables were described as percentages and compared using chi-squared tests. For patients with EC and the control group, APN and hs-CRP levels were measured; anthropometric data were obtained during a chart review. Statistical comparisons between groups were analyzed using Student’s *t*-test; correlations were confirmed using the Pearson correlation. The risk of AF was calculated by multivariable Poisson regression analysis and the incidence rate ratio (IRR). The risk of AF was calculated by Cox proportional hazard regression, the hazard ratios (HRs), and 95% confidence interval, using normal BMI as the reference group and as Model 1 adjusted for age; Model 2 adjusted for drinking, smoking, and BMI; and Model 3 adjusted for history of hypertension, diabetes mellitus, hyperlipidemia, past myocardial infarction (>3 months ago), and hs-CRP. Additionally, considering the rule of frequency-matching design, we also introduced the age and covariates information variables. Then, stepwise regression was used to establish a regression equation. In secondary analyses, we calculated the statistical significands between APN, hs-CRP, and AF. Statistical analysis was carried out using the SPSS v.17 statistical software (SPSS Inc., Chicago, IL, USA). A two-sided *p*-value <0.05 was considered significant.

## 3. Results

### 3.1. The Study Cohort

A total of 515 postmenopausal Bulgarian women with vaginal bleeding and newly diagnosed, histologically verified EC (case group, *n =* 310) or with normal (senile) endometrium or endometrium with hyperplasia (control group, *n =* 205) were registered before any treatment with a baseline visit in a Department of Obstetrics and Gynecology between 2014 and 2017 (Figure 1). As seen in Table 1, the mean age of patients with EC in this study was 63.34 ± 7.03, while the mean age of individuals in the control group was 65.17 ± 6.16 (*p* = 0.273). The age distribution in EC patients and the control population younger than 60 years was different, with EC patients being younger than the control population (Table 1). The mean BMI value for patients with EC and the control group was 31.04 ± 4.96 kg/m^2^ and 28.62 ± 3.04 kg/m^2^, respectively (*p* = 0.059). As presented in Figure 1, 15 patients were excluded from the cohort due to a pre-existing diagnosis of AF before their baseline visit. Another 11 patients declined to participate. The remaining 489 patients (*n =* 298, 60.9% with EC by histology and *n =* 191, 39.1% with normal endometrium/endometrial hyperplasia) were included in the study analysis. Follow-up was completed with a mean time of 2.5 years (SD ± 0.5 years). The median time from the diagnosis of EC to a diagnosis of AF was 188 days (interquartile range 93–398). Women with EC did not present with a significantly higher prevalence of hypertension, diabetes, and hyperlipoproteinemia. Significant differences in risk factors such as current smoking and alcohol consumption were observed in women with EC compared to women without EC. Patient characteristics stratified by EC status are presented in Table 1.

### 3.2. Incidence of AF

During the mean follow-up of 2.5 ± 0.5 years, new-onset AF was detected in 35/298 (11.7%) women with EC. In the control group (*n =* 191) with normal endometrium/endometrial hyperplasia, 11 new cases of AF were detected (5.8%). The incidence of AF stratified by age was 41.7 (95% confidence interval (CI) 27.1–63.5) for those patients with EC aged <60 years, and the incidence of AF in the control population of the same age was 17.5 (95% CI 17.4–20.1). The incidence of AF in patients with EC and older than >60 years was 60.9 (95% CI 55.5–78.3) vs. 26.3 (95% CI 25.5–36.7) in the control group.

### 3.3. Risk of AF

The IRR of AF in the EC cohort was calculated with multivariable Poisson regression analysis and was 1.27 (95% CI 1.17–1.35; *p* < 0.01) compared to subjects with normal endometrium/endometrial hyperplasia. The risk of AF was significantly increased within the first 6 and 12 months after the EC diagnosis, with an IRR of 1.19 (95% CI 1.10–0959; *p* = 0.01) and 1.16 (95% CI 1.07–1.28; *p* = 0.0037), respectively. AF does not exist in isolation, and it always occurs in combination with other morbidities [3]. Therefore, the analysis of cardiovascular risk factors within the EC population revealed the association of hypertension with an increased risk of AF, with an IRR of 1.31 (95% CI 1.23–1.47; *p* = 0.025), while no association was found for diabetes, with an IRR of 0.93 (95% CI 0.67–1.28; *p* = 0.753). Regarding the pharmacological treatment of patients with EC such as chemo- and hormone therapy, no association was demonstrated between the risk of AF and medical treatment. Drug treatments for EC include chemotherapy such as paclitaxel; carboplatin; doxorubicin; or liposomal doxorubicin, cisplatin, and docetaxel. The main hormone treatment for EC uses progesterone or drugs similar to it (called progestins).

### 3.4. Assessment of Other Covariates with New-Onset AF

Information on other covariates was collected during a physical examination at baseline (Table 1). Smoking status, alcohol intake, and the use of antihypertensive medication were self-reported. At baseline, participants’ BMI was categorized as normal (18.5 to 24.9 kg/m^2^), overweight (25 to 29.9 kg/m^2^), and obese (>30 kg/m^2^). HRs for AF using BMI in the total population are shown in Table 2. Using normal BMI (18.5 to 24.9 kg/m^2^) as the reference group, we found that multivariable-adjusted HRs (95% CI) for AF were 0.94 (0.49–1.79), 1.0 (ref), and 1.80 (1.01–3.02) from the lowest to the highest category of BMI, respectively. The new onset of AF in women with EC who were overweight or obese was more likely, while this relationship was not significant among women with normal endometrium/endometrial hyperplasia (HR 1.69; 95% CI 0.70–4.11; *p* = 0.75). Further analysis with stratification by different age groups was used (Table 2). Patients with EC aged <60 years with a BMI of >30 kg/m^2^ were found likely to develop AF (HR 1.95; 95% CI 1.18–3.32), while this relationship was not significant among patients aged ≥60 years (HR 1.22; 95% CI 0.83–1.88; Table 2). We further repeated the analysis in the control group. Overweight or obesity in participants without EC in either age group (<60 years and ≥60 years) showed no association with the development of AF (Table 2). In our study, LA size was significantly associated with an increased risk of AF, with an IRR of 1.39 (95% CI 1.15–1.50; *p* = 0.04) after adjusting for obesity within the EC population. Age- and sex-adjusted risk of AF increased with higher BMI and larger LA diameter.

### 3.5. Adiponectin and AF Risk

To investigate the reason for the increased AF risk in EC women and assess the impact of putative mediators, we conducted a secondary analysis and entered specific covariates that might explain the causal pathway between EC and AF. The mean APN concentrations in patients with EC were significantly lower than those of the control group (2.79 ± 0.66 vs. 5.15 ± 2.63 ng/mL, respectively; *p* = 0.007). However, there was no significant difference in APN levels between type I EC and type II EC (data not shown). There was no evidence for a connection between APN plasma levels alone and the risk of AF, but a tendency was observed (95% CI 0.87–1.21; *p* = 0.063). BMI in women with EC (31.04 ± 4.96) tended to be higher (not statistically significant) compared to the control group (28.62 ± 3.04). Furthermore, a significant correlation was identified between APN, BMI, and EC (*R* = 0.373; *p* < 0.001) and evidence of APN–AF association was observed when adjusted for BMI (2.05; 95% CI 1.04–4.04; *p* = 0.037). Moreover, after adjusting for standard AF risk factors, including age, diabetes, hypertension, past myocardial infarction, dyslipidemia, smoking, alcohol consumption, and hs-CRP, there was no evidence of significant interaction. In a secondary analysis, BMI ≥30 kg/m^2^ combined with lower APN plasma levels showed a significantly increased risk of AF (HR 2.05; 95% CI 1.04–4.04; *p* = 0.037). Overall, the level of hs-CRP was statistically higher in women with EC compared to control women (10.80 ± 1.7 vs. 0.80 ± 0.3 mg/L, respectively; *p* = 0.009). However, there was no evidence of a connection between hs-CRP alone and the risk of AF (*p* = 0.45). In a secondary analysis, BMI ≥ 30 kg/m^2^ combined with elevated hs-CRP levels showed a significantly increased risk of AF (HR 2.09; 95% CI 1.07–4.07; *p* = 0.041). A significant difference across BMI dimensions was found for APN (<0.01) and hs-CRP (<0.001) after dividing the study population according to three dimensions of BMI (Table 3). Moreover, APN inversely correlated with three BMI dimensions and hs-CRP levels (Table 3).

## 4. Discussion

Many of the risk factors of EC overlap with those for cardiovascular diseases such as coronary artery disease, HF, and AF [23,24]. Notably, women represent a population at a higher risk of severe cardiac and arrhythmic complications [25,26]. Despite all these relevant issues, we need further investigations to define better the mechanisms underlying these gender-related differences and gender-specific recommendations in the current guidelines for screening and therapy for cardiac and arrhythmic disorders [27]. Therefore, we hypothesized that there could be an association between EC and risk of AF. These findings could have a potential application for identifying gender-specific populations at a high risk of AF.

In this prospective, single-center study with a relatively large cohort of 310 women with EC, we report an association between EC and an increased risk of AF after multivariable adjustment for clinical risk factors compared with the age-adjusted control population with non-cancerous (normal and/or hyperplastic) endometrium (Table 1). During mean follow-up of 2.5 ± 0.5 years, new-onset AF had occurred in 11.7% women with EC vs. 5.8% in the control group (*p* < 0.01). Furthermore, we performed sensitivity analyses adjusting for measures of total body size to validate and explore the association between BMI and AF in postmenopausal women with EC (Table 2). In a secondary analysis, overweight (BMI > 25 kg/m^2^) and obese (BMI ≥ 30 kg/m^2^) women with EC were significantly associated with a higher risk of AF (Table 2). This relationship persisted independently of other known major risk factors for AF, such as diabetes, hypertension, past myocardial infarction, dyslipidemia, smoking, and alcohol consumption (Table 2). Furthermore, the median time of 6 months between the diagnosis of EC and the diagnosis of AF indicated a persisting risk of AF after the first intensive treatment associated with the evaluation of EC in a hospital setting.

Both inflammation and obesity are risk factors for AF, and adipose tissue is a known contributor to systemic inflammation. APN is adipokine expressed by fatty tissue, and it has been associated with multiple known risk factors for AF, including diabetes, obesity, inflammation, and HF [28,29]. Reduced APN levels are linked to insulin resistance, obesity, and metabolic syndrome [30,31]. However, the predictive value of APN in cardiovascular disease has been reported to be contradictory, and its relationship with AF has been controversially discussed [26,27,28,29]. Although fat mass considerably increases in obesity, APN concentration is strongly reduced in obese patients due to this tissue’s chronic inflammation [32]. Compared to baseline levels, adipose tissue functions improves after weight loss, and serum APN levels increase [32]. We performed a prospective study with histologically confirmed EC and control women admitted during the same period. In the present study, we investigated biomarkers that may mediate the association between EC and AF risk. We found a moderately strong relationship between circulating levels of APN, hs-CRP, and BMI in patients with AF and EC (Table 3).

Indeed, adjustment for BMI, but not for the other standard AF risk factors, showed significant association (HR 2.05; 95% CI 1.04–4.04; *p* = 0.037). Our current investigation found that both APN and hs-CRP were independently associated with AF, even after adjustment for BMI, showing an association between these biomarkers and AF in postmenopausal women with EC. Women with EC at high risk of AF had low levels of APN and high levels of hs-CRP. Because these inflammatory biomarkers were measured at baseline in a cohort of individuals without known AF, these findings could strongly support the hypothesis that chronic inflammation contributes to the clinical setting of AF in postmenopausal women with EC.

Another finding of the present study is the inverse relationship between APN and hs-CRP indicating that the risk of AF is increased when hs-CRP is elevated and APN is low (Table 3). The inverse correlation between APN and hs-CRP may provide novel insights on in vivo inflammation activation in patients with EC. Interestingly, APN was also inversely related to three BMI dimensions (Table 3). Because chronic inflammation and neurohormonal activation are established etiological factors for both conditions, EC and AF, it could be supposed that inflammation is the major component in the increased risk of new-onset AF in postmenopausal women with EC [8,9]. Furthermore, it was observed that other diseases with an inflammatory genesis such as type II diabetes are characterized by an increased risk of development of AF [31,32,33].

In conclusion, the present study shows that women with EC at high risk of AF displayed low and high levels of APN and hs-CRP, respectively, suggesting a role for APN in chronic inflammation activation in vivo in the clinical setting of EC. Reducing BMI and enhancing APN levels may be a future goal to reduce the risk of AF in postmenopausal women, especially younger (<65 years) women, with EC. The substantial proportion of the EC and AF association mediated by inflammation adds to the understanding of AF pathogenesis and could have important treatment implications. In general, more studies are hence needed to confirm the putative relationship between the gender-specific hormone profile patterns and AF risk.

Furthermore, our findings show that inflammation is a more prominent and important etiological factor for new-onset AF in women with EC. Moreover, from a clinical standpoint, these results suggest that interventions targeting inflammation status may be the most important to modulate and decrease the risk of AF. Indeed, EC-associated AF may be a special kind of atrial disease subtype, requiring customized therapy.

### Strengths and Limitations

This study has limitations, which should be mentioned. On the basis of this study, we were unable to establish an etiological relationship between inflammation and AF, and inflammation suppression as preventive therapy for AF. In our study, AF was diagnosed on the basis of a single ECG without ambulatory ECG monitoring, and the EC patients had more frequent episodes of healthcare. For the detection of short phases of AF, more extensive monitoring techniques such as 24-h Holter monitoring, 7-day Holter monitoring, 10-day Zio Patch monitoring, and even continuous monitoring with modern implantable loop recorders should be considered in future studies. The combination with AF can be permanent but is often paroxysmal in the beginning. Within the scope of this study, only individual ECGs were documented, and no distinction was made between paroxysmal and non-paroxysmal atrial fibrillation. We investigated only the association between EC and future AF at baseline examination, recording baseline blood chemistry and a minimal number of biological variables, without considering changes of the blood status and general health of study participants. Indeed, any subsequent change in blood chemistry could lead to non-differential misclassification and potentially underestimate the EC–AF association. This study solely considered APN rather than the full range of adipokines that could affect the risk of EC development. Another limitation is that candidate controls were selected through convenience sampling. Finally, our results may not be generalizable to other races/ethnicities or younger individuals. Our findings should be interpreted with caution as they constitute a pilot study rather than a definitive one.

## 5. Conclusions

In conclusion, the present results suggest that postmenopausal women with EC carry an increased risk of AF, compared with the general population with non-cancerous (normal and/or hyperplastic) endometrium. Furthermore, serum APN plays an important role in the development of obesity-related EC. Nevertheless, increased awareness of early signs of AF is warranted among EC patients to attain earlier diagnosis and treatment, since the risk remains increased several months after the EC diagnosis. Furthermore, the cardiac risk profile should be drawn up and close monitoring should be carried out during survivorship [24,34]. It is deemed necessary to clarify the role of obesity in endometrial carcinogenesis, developing a strategy to combat obesity and the development of a non-invasive diagnostic test to evaluate the risk of AF in postmenopausal women with EC.

## Figures and Tables

**Table 1:** Baseline characteristics of study participants.

Parameters	Control Group (*n* = 205)	Case Group (*n* = 310)	*p*-Value
Age, years	65.17 ± 6.16	63.34 ± 7.03	ns
BMI, kg/m2	28.62 ± 3.04	31.04 ± 4.96	ns
Systolic blood pressure, mmHg	128 ± 5	131 ± 10	ns
Diastolic blood pressure, mmHg	83 ± 4	85 ± 6	ns
Total cholesterol, mmol/L	5.11 ± 0.8	5.90 ± 1.0	ns
Triglycerides, mmol/L	1.63 ± 1.4	1.70 ± 1.7	ns
LDL, mmol/L	3.3 ± 0.5	4.1 ± 0.7	ns
HDL, mmol/L	1.5 ± 0.6	1.4 ± 0.5	ns
Fasting blood glucose, mmol/L	5.4 ± 1.6	5.5 ± 1.8	ns
Past myocardial infarction, n (%)	29 (15.2)	41 (13.8)	ns
Current smoker, n (%)	100 (52.4)	167 (56)	**
Current alcohol, n (%)	51 (26.7)	93 (31.2)	**
APN, ng/mL	5.15 ± 2.63	2.79 ± 0.66	**
hs-CRP, mg/L	0.80 ± 0.3	10.80 ± 1.7	**
Serum creatinine, mg/dL	0.80 ± 0.7	0.85 ± 1.3	ns
Thyroid-stimulating hormone, mU/L	2.97 ± 1.67	3.97 ± 1.07	ns
Mean LA diameter (cm)	4.0 ± 0.9	4,5 ± 0.7	*
Low LVEF (<50%), n (%)	10 (4.9)	19 (6.1)	ns

Values are mean ± SD. Data were compared, and *p*-values are calculated by Kruskall–Wallis test and indicate the difference between a control group and case subjects with EC. Categorical variables were described as percentages and compared using chi-square tests. * *p* < 0.05, ** *p* < 0.01 and ns (not significantly) versus control group.

**Table 2:** Hazard ratios (HRs) (95% CI) for atrial fibrillation (AF) according to body mass index (BMI) stratified by age in the endometrial cancer (EC) (**a**) as well as in the non-EC (**b**) group.

(**a**)				
**EC**	**BMI (kg/m2)**			**p-Value**
	**Overweight25 to 29.9**	**Normal18.5 to 24.9**	**Obese(>30)**	
**Age < 60**				
Model 1	0.99 (0.55–1.73)	reference	1.95 (1.18–3.32)	0.05
Model 2	1.21 (0.89–1.85)	reference	1.86 (1.09–3.18)	0.04
Model 3	0.94 (0.69–1.79)	reference	1.80 (1.01–3.02)	0.05
**Age ≥ 60**				
Model 1	1.17 (0.71–1.72)	reference	1.22 (0.83–1.88)	ns
Model 2	1.01 (0.72–1.67)	reference	1.30 (0.84–2.00)	ns
Model 3	1.0 (0.71–1.70)	reference	1.27 (0.84–1.91)	ns
(**b**)				
**No EC**	**BMI (kg/m2)**			**p-Value**
	**Overweight25 to 29.9**	**Normal18.5 to 24.9**	**Obese(>30)**	
**Age < 60**				
Model 1	1.19 (0.54–1.84)	reference	1.95 (1.34–2.92)	0.10
Model 2	1.00 (0.54–1.85)	reference	1.95 (1.20–3.50)	0.09
Model 3	0.95 (0.51–1.78)	reference	1.86 (1.09–3.23)	0.14
**Age ≥ 60**				
Model 1	1.13 (0.74–1.77)	reference	1.37 (0.79–1.75)	ns
Model 2	1.10 (0.82–1.77)	reference	1.25 (0.81–1.92)	ns
Model 3	1.10 (0.81–1.95)	reference	1.30 (0.94–2.00)	ns

Model 1: Adjusted for age. Model 2: Adjusted for drinking, smoking, and BMI. Model 3: Adjusted for history of hypertension, diabetes mellitus, hyperlipidemia, past myocardial infarction (>3 months ago), and high-sensitivity C-reactive protein-(hs-CRP).

**Table 3:** Median values of adiponectin (APN) and hs-CRP according to BMI.

	BMI (kg/m^2^)			*p*-Value
	Normal18.5 to 24.9	Overweight25 to 29.9	Obese(>30)	
APN, ng/mL	7.1 (5.3–8.7)	5.5 (4.9–7.0)	3.7 (2.8–4.5)	<0.01
hs-CRP, mg/L	0.7 (0.5–1.7)	5.9 (5.5–8.9)	11.70 ± 2.17	<0.001

## Data Availability

The data presented in this study are available on request from the corresponding author. The data are not publicly available due to ethical restrictions.
